# Intergenerational Relationships During the COVID-19 Pandemic: Challenges and Opportunities of Data Collection

**DOI:** 10.1093/geroni/igab046.644

**Published:** 2021-12-17

**Authors:** J Jill Suitor, Megan Gilligan

**Affiliations:** 1 Purdue University, Purdue University, Indiana, United States; 2 Iowa State University, Iowa State University, Iowa, United States

## Abstract

Family scholars experienced numerous unique challenges and opportunities when studying intergenerational relationships during the COVID-19 pandemic. In this presentation, we draw examples from the Within-Family Differences Study to highlight some of the ways in which respondents’ patterns of study participation, reports of their relationships with their late-life parents and their own young adult children, and their psychological well-being, subjective physical health, and health behaviors reflected the fluctuating waves of the pandemic. Among the patterns we discuss are systematic variations in the intensity of respondents’ answers to both open and closed-ended items, respondents’ expressions of concern regarding choosing the “right” answers to questions, and their attempts to negotiate their responses with interviewers. Our observations led us to conclude that measures family gerontologists use to capture many constructs central to the field are subject to different “meanings” by respondents in the face of disaster.

